# Evaluation of the London Smoking Cessation Transformation Programme: a time–series analysis

**DOI:** 10.1111/add.15367

**Published:** 2020-12-29

**Authors:** Sarah E. Jackson, Emma Beard, Robert West, Jamie Brown

**Affiliations:** ^1^ Department of Behavioural Science and Health University College London London UK; ^2^ SPECTRUM Consortium London UK

**Keywords:** Quit attempts, quit success, smoking, smoking cessation, stop‐smoking services, time–series analysis

## Abstract

**Background and aim:**

National social marketing campaigns have been shown to promote smoking cessation in England. There is reason to believe that regional and city‐wide campaigns can play a valuable role in reducing smoking prevalence over and above any national tobacco control activity. This study aimed to assess the impact of the London Smoking Cessation Transformation Programme, a multi‐component citywide smoking cessation programme, on quit attempts and quit success rates.

**Design and Setting:**

Interrupted time–series analyses, using Autoregressive Integrated Moving Average (ARIMA) and generalized additive models (GAM) of population trends in the difference between monthly quit attempts and quit success rates among smokers who made a quit attempt in London versus the rest of England before and during the first year of the programme.

**Participants:**

A total of 55 528 past‐year adult smokers who participated in a monthly series of nationally representative cross‐sectional surveys in England between November 2006 and August 2018. Twelve and a half per cent of smokers lived in London (intervention region) and 87.5% lived in the rest of England (control region).

**Measurements:**

Monthly prevalence of quit attempts and quit success rates among smokers who made a quit attempt.

**Findings:**

The monthly difference in prevalence of quit attempts in London compared with the rest of England increased by 9.59% [95% confidence interval (CI) = 4.35–14.83, *P* < 0.001] from a mean of 0.04% pre‐intervention to 9.63% post‐intervention. The observed increase in success rates among those who tried was not statistically significant (*B* = 4.72; 95% CI = –2.68 to 12.11, *P* = 0.21); Bayes factors indicated that these data were insensitive. GAM analyses confirmed these results.

**Conclusion:**

The promotion of the London Smoking Cessation Transformation Programme during September 2017 was associated with a significant increase in quit attempts compared with the rest of England. The results were inconclusive regarding an effect on quit success among those who tried.

## Introduction

Tackling smoking remains a public health priority in England [1], as in many other countries [2]. There is reason to believe that regional and citywide campaigns can play a valuable role in reducing smoking prevalence over and above any national tobacco control activity [3, 4]. In 2017, a city‐wide smoking cessation campaign was launched in London to boost quitting rates. The campaign involved use of mass media and on‐line marketing, an on‐line portal and a dedicated telephone helpline. This study examined whether the campaign resulted in an increase in quit attempts and in the success rate among smokers who tried to quit in the first year of operation.

In England, smoking prevalence and stop smoking service provision vary substantially by local authority district, both within and outside London [5, 6]. Since 2013, local authorities have held responsibility for commissioning stop smoking services and Government cuts have seen budgets for local stop smoking services decline by almost a third (£41.3 million since 2014/15) [6]. As a result, the local offer of support and advice to smokers has diversified. In 2018, just over half (56%) of local authorities offered a universal stop smoking service to all local smokers; 22% commissioned an integrated life‐style service instead of a stop smoking service, 9% commissioned stop smoking support in primary care only and 3% did not commission any services [6]. A recent report by Cancer Research UK and Action on Smoking and Health emphasized the importance of local innovation in smoking cessation service delivery in the context of ongoing cost constraints [6]. There has also been a decline in central funds dedicated to tobacco control. In making an urgent case for a new tobacco fund through the polluter pays principle, the SmokeFree Action coalition describe how budgets for mass media (public education) campaigns (which have been shown to be effective in increasing quitting activity [7, 8, 9, 10, 11, 12]) have been reduced substantially in England in recent years [13].

Work to develop the London Smoking Cessation Transformation Programme (LSCTP) began in November 2016, with the aim of supporting London local authorities to transform and improve smoking cessation across the capital [14]. This followed a sector‐led improvement review involving 32 London local authorities in 2014–15 [15, 16]. The programme was launched with a social marketing campaign in September 2017 to direct smokers in the capital to a London‐wide Stop Smoking helpline and an on‐line portal providing information on smoking, the benefits of stopping and directing smokers to available support services in their area [17]. The campaign included radio and on‐line advertising of both the helpline and on‐line portal, and was coordinated with national campaigns, including Stoptober [18] and No Smoking Day [19]. This approach is novel, in that the focus is on providing a city‐wide portal to free specialist cessation services, backed up by telephone support for those smokers who want it. This is only possible in countries or cities that have such services and means that the media campaigns can focus upon directing people to receipt of cessation support rather than the more conventional health harms approach or a general call to action. The international relevance of this London‐based intervention is a demonstration that media campaigns and cessation support can work together to improve quitting rates in large population groups. Further details of the LSCTP can be found in the Supporting information.

Given that the LSCTP is a multi‐component programme aimed at promoting quit attempts and increasing their chances of success, it is important to evaluate both of these outcomes separately. The Smoking Toolkit Study (STS) provided a means of conducting this. It involves a monthly series of national household surveys that began in November 2006. The extensive time–series generated should provide a basis for detecting an increase attributable to the LSCTP in London versus the rest of the county from September 2017 to August 2018. The STS asks separately about quit attempts and quit success [20]. To our knowledge, there was no other London‐specific tobacco control activity initiated at the same time as the LSCTP that could act as a confounding factor.

If the LSCTP increases quitting activity, it could provide a blueprint for similar programmes in other major cities in the United Kingdom or overseas. It is also important to be able to estimate its effect in London to assess whether to continue to fund it. Thus, this study aimed to evaluate, using time–series analysis, whether the LSCTP resulted in a detectable increase in quit attempts and the success of those attempts in its first year of operation.

## Method

### Intervention: on‐line portal and helpline

The LCSTP was a new programme that was set up specifically for the purpose of increasing quitting rates in London, with a budget of £450 000. No other parts of the country had a comparable programme, but others had programmes that had been in place for longer focusing upon wider tobacco control measures and coordination of services (e.g. FRESH North East).

The Stop Smoking London on‐line portal provided evidence‐based information about the reasons for smoking, tools and resources to help smokers to quit, local support services and the benefits of stopping smoking in an interactive, user‐friendly format. The helpline number featured prominently at the top of each page, and users could search by borough or postcode to locate and access local support.

The helpline was a national service provided by NHS Smokefree to smokers across England. The local rate number was available 7 days a week—Monday to Friday from 9 a.m. to 8 p.m. and on Saturday and Sunday between 11 a.m. and 4 p.m. Professionally trained advisers advised on locally available support and which quit methods worked best, including different types of medication, mobile applications (apps) and specialist programmes. Smokers were asked a series of questions to determine the level of stop smoking support required. Those from London and who were not pregnant were offered the opportunity to sign up to a specialist telephone‐based service, which provided a proactive 4‐week behavioural support intervention.

The Stop Smoking London programme differed from other national stop smoking services in two key ways. First, it provided a targeted marketing strategy designed to attract London smokers to the service. Once a smoker made contact, the aim was for the service to promptly and conveniently triage them appropriately into local and regional support, according to their individual need. Secondly, the 4‐week proactive behavioural support helpline that Stop Smoking London offered was exclusively available for Londoners. These differences in stop smoking service offerings between London and the rest of England did not exist prior to the development of the LSCTP: the services varied across local authorities in London as they did in the rest of the country, with no significant different across regions.

Different components of the programme were available from May 2017, but the first time they were all promoted was September 2017.

### Ethics approval and consent to participate

Ethical approval for the STS was granted originally by the UCL Ethics Committee (ID 0498/001) and participants provided full informed consent. The data are not collected by UCL and are anonymized before being received by UCL.

### Availability of data and materials

The data and commands used to analyse them are available on Open Science Framework (https://osf.io/drcv9/).

### Study population

Data were drawn from the ongoing STS, a monthly cross‐sectional survey of a representative sample of adults (≥ 16 years) in England that monitors trends in a range of variables relating to smoking [20]. The study uses a form of random location sampling to select a new sample of approximately 1700 adults (450 smokers) aged ≥ 16 years each month. The survey typically covers 200–300 census output areas each wave, which are sampled at random (after stratification by geodemographic analysis of the population) from more than 170 000. Interviewers travel to the selected areas and perform computer‐assisted interviews with one participant aged over 16 years per household until quotas based upon factors influencing the probability of being at home (working status, age and gender) are fulfilled. Random location sampling is considered superior to conventional quota sampling because the choice of properties approached is reduced by the random allocation of small output areas. However, interviewers can still choose which houses within these areas are most likely to fulfil their quotas, rather than being sent to specific households in advance. Response rates are therefore not appropriate to record, unlike random probability sampling, where interviewers have no choice as to the properties sampled and so response at each address can be recorded, Comparisons with national data and retail sales indicate that key variables such as socio‐demographics and smoking prevalence are nationally representative [20, 21].

For the present study, we used monthly data from respondents to the survey in the period from November 2006 (the first wave of the STS) to August 2018 (1 year after the campaign started). The STS has been used successfully to evaluate a number of national tobacco control initiatives [18, 19, 22, 23, 24].

### Patient and public involvement

During the planning phase of the LSCTP, the programme organizers undertook research and insight work with key population groups and stakeholders across London, in order to: (i) improve understanding of the programme's target audience, their motivations for smoking and their motivations for giving up smoking, and (ii) inform the design and delivery of a behaviour change campaign that encourages the target audience to seek and use London‐wide stop smoking services.

The wider toolkit study has been discussed with a diverse patient and public involvement (PPI) group and the authors regularly attend and present at meetings at which patient and public are included. Interaction and discussion at these events help to shape the broad research priorities and questions. Members of the public were not involved in setting the specific research questions or the outcome measures, or in the design and implementation of this specific study.

### Measures

The independent variable was government office region in England, dichotomized to London versus other (North East, North West, Yorkshire and the Humber, East Midlands, West Midlands, East of England, South East, and South West).

Outcome variables were monthly prevalence of (i) quit attempts and (ii) quit success rates among those who tried. These were assessed via two questions asked of past‐year smokers in each STS wave:

1. How many serious attempts to stop smoking have you made in the last 12 months? By serious attempt I mean you decided that you would try to make sure you never smoked again. Please include any attempt that you are currently making and please include any successful attempt made within the last year.
2. How long did your most recent serious quit attempt last before you went back to smoking (still not smoking/less than a day/less than a week/more than 1 week and up to a month/more than 1 month and up to 2 months/more than 2 months and up to 3 months/more than 3 months and up to 6 months/more than 6 months and up to a year)?

The prevalence of quit attempts in each month was calculated as the number of respondents who reported having made one or more quit attempts in the past 12 months divided by the number of past‐year smokers. The success rate in each month was calculated as the number of respondents reporting that they were still not smoking divided by the number who reported having made a quit attempt. An advantage of this measure of success over requiring a certain duration of abstinence (e.g. 6 months) is that it has a clear relationship to the quit attempt in question and minimum demand for recall (a participant only has to remember whether they are currently not smoking and whether their attempt began in the last year) [25, 26]. The overall quit rate was calculated as the number of respondents reporting that they were still not smoking divided by the number of past‐year smokers.

We had intended to analyse use of cessation support as a third outcome, but the sample of London‐based smokers was too small. However, we provide descriptive data on use of cessation support [any of face‐to‐face behavioural support, prescription medication, telephone support or digital (websites, apps) support: coded 1 for any, 0 for none] before and after the intervention in London compared with the rest of England.

We also recorded data on participants' age, sex and social grade (ABC1, which includes managerial, professional and intermediate occupations, versus C2DE, which includes small employers and own‐account workers, lower supervisory and technical occupations and semi‐routine and routine occupations, never worked and long‐term unemployed) for inclusion as covariates in some analyses. This occupational measure of social grade is a valid index of socio‐economic status (SES) that is widely used in research in UK populations and has been identified as particularly relevant in the context of tobacco use and quitting [27] and other addictive behaviours [28].

### Statistical analysis

The analysis plan was pre‐registered on the Open Science Framework (https://osf.io/drcv9/). We made two modifications to the pre‐registered plan: (i) not analysing use of cessation support, due to insufficient data, and (ii) including past‐month quit attempts and the overall quit rate as additional outcomes. Our rationale for adding past‐month quit attempts as a sensitivity analysis was if all the outcome measures relied upon quit attempts in the last year, the assessment period after the intervention would not have related exclusively to the intervention. Our rationale for adding an analysis of the overall quit rate was that this would combine quit attempts and success and provide a direct estimate of the impact of the intervention on cessation. All data were analysed in R [29].

For the primary analyses, data were aggregated monthly and weighted to match the population in England [20]. The independent variable was a variable coded 0 until, but not including, September 2017 and then 1 until August 2018 inclusive, reflecting the first year of implementation of the programme. The dependent variable was the difference in prevalence of (i) quit attempts and (ii) quit success rates between London (intervention region) and the rest of England (control region). Where any prevalence values were zero (*n* = 18 data points), values were imputed using Kalman smoothing for univariate time series data [30] (see [31, 32] for a detailed introduction to Kalman filtering). Results are reported with and without imputation. As an unplanned third outcome, we analysed the overall quit rate among smokers (i.e. the proportion of all smokers who successfully quit).

Autoregressive integrated moving average (ARIMA) analysis was used to estimate the impact of the intervention on attempts to quit smoking and the success of attempts to quit smoking [33, 34, 35]. The *B* coefficients can be interpreted as the total change in the series mean from the pre‐ to the post‐intervention period attributable, in the absence of confounding, to the intervention. Standard recommended procedures [33, 36] were used to select the ARIMA models (see Supporting information for details). The best‐fitting model for the analyses was an ARIMA (0,1,1)_12_, i.e. a model with one order of differencing and one non‐seasonal MA term. Models were also run with the addition of a seasonal AR term, i.e. a seasonal ARIMA (0,1,1) (1,0,0)_12_, because model residuals were not free of serial correlation. A sensitivity analysis was conducted which forecast the predicted prevalence of quit attempts and quit success from September 2017 using the pre‐intervention data. This gives the predicted values for September 2017 until August 2018, assuming that no intervention had taken place.

A secondary difference‐in‐differences (DID) sensitivity analysis was then conducted using generalized additive models (GAMs) at the individual non‐aggregated level. These models allow the fitting of seasonal smoothing terms, and therefore allow seasonality to be taken into account. The DID model unadjusted for sex, age, and social grade was specified as: y_t_ = β_0_+β_1_trend+β_2_level_t_+β_3_slope+β_4_trend × intervention+β_5_ × intervention+β_6_slope × intervention+e_t_, where trend is a variable coded 1 … *n* (*n* is the total number of time‐points to the end of the series) reflecting the time trend over time, and level is a dummy variable coded 0 before the intervention and 1 after to reflect a step‐level change. Finally, slope is a variable coded 0 before the event and 1 … *n* after and reflects the change in the pre‐intervention slope post‐intervention. An adjusted model is also reported adjusting for age, sex and social grade.

Our rationale for using both ARIMA and GAM models was that each offers distinct benefits: the ARIMA is a ‘true' time–series approach that uses past and current values in the prediction, while the GAM can detect changes in trend beyond the enduring step change modelled in the time–series.

We calculated Bayes factors (BF; planned a priori) for non‐significant results in order to examine whether those findings could best be characterized as evidence of no effect or whether data were insensitive to detect an effect [37, 38]. Alternative hypotheses were represented by half‐normal distributions and the absolute expected effect size for ARIMA results was set to *B* = 0.75 and for GAM results was set to odds ratio (OR) = 1.06. These expected effect sizes were based on a previous study that reported a 0.51% increase in quit success for every 10% increase in tobacco control mass media campaign expenditure [39]. BFs ≥ 3 can be interpreted as evidence for the alternative hypothesis (and against the null), BFs ≤ 1/3 as evidence for the null hypothesis, and BFs between 1/3 and 3 suggest the data are insensitive to distinguish the alternative hypothesis from the null [37, 40].

## Results

Data were collected from 55 528 past‐year smokers. Data from these past‐year smokers were aggregated monthly and stratified by region (London versus the rest of England) between November 2006 and August 2018 inclusive. This included a sample of 6950 smokers in London [12.5% of the total analysed sample; smoking prevalence in London 18.7%, 95% confidence interval (CI) = 18.3–19.1] and 48 577 smokers in the rest of England (87.5% of the total analysed sample; smoking prevalence in the rest of England 22.8%, 95% CI = 22.6–22.9).

**Figure 1:**
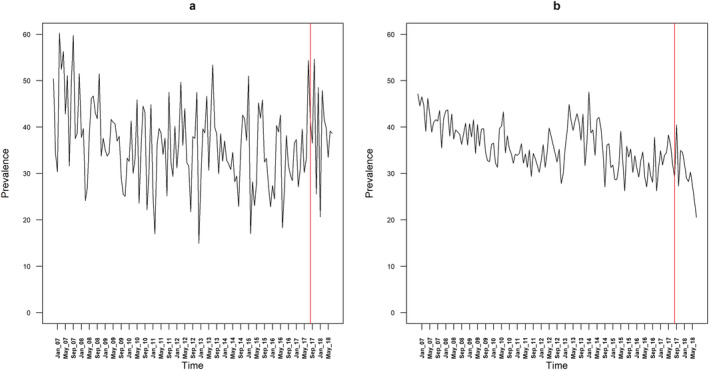
Prevalence of quit attempts in (a) London and (b) the rest of England. The red line denotes the time at which the intervention was launched

**Figure 2:**
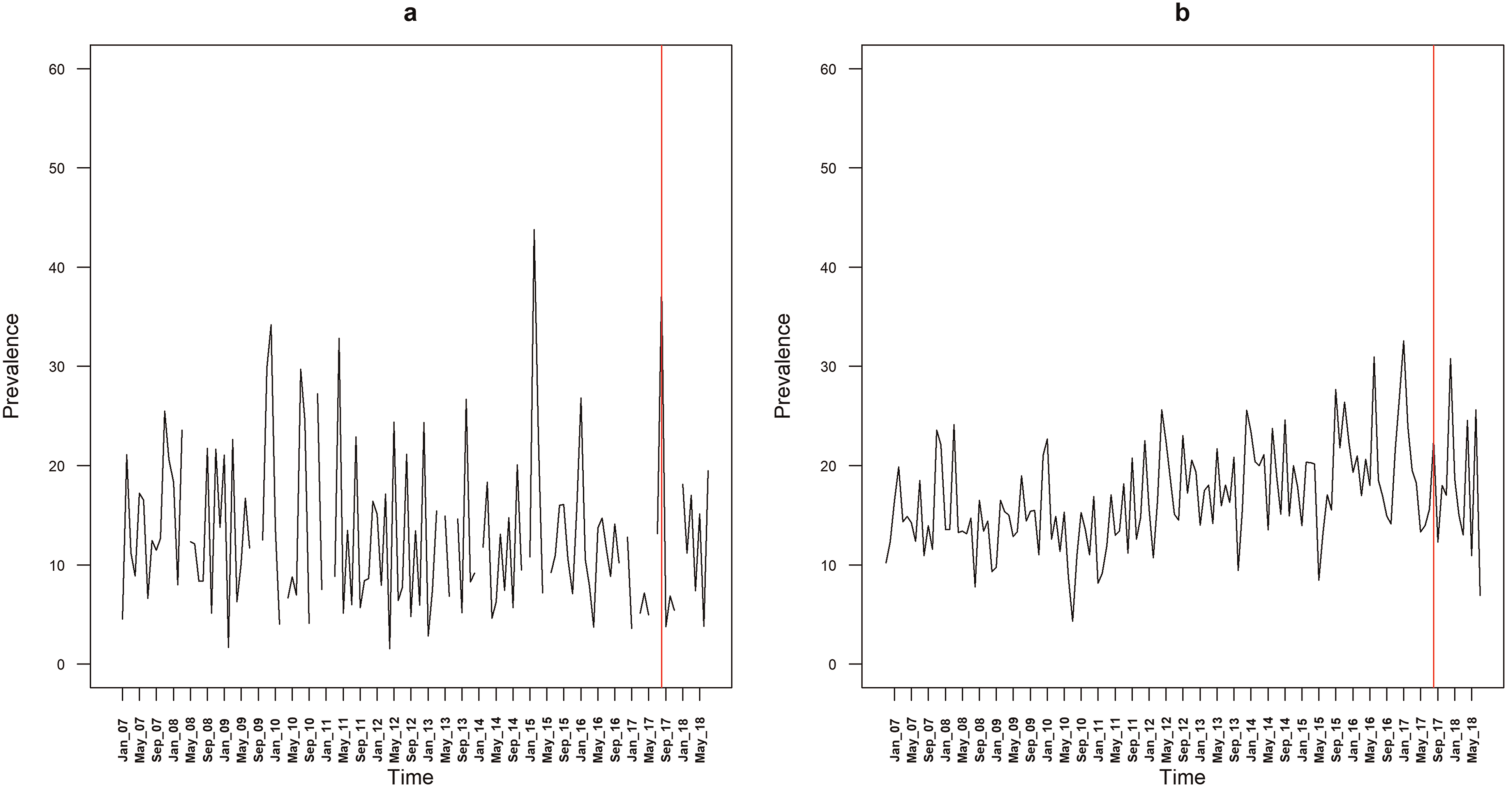
Success rate of quit attempts in (a) London and (b) the rest of England. The red line denotes the time at which the intervention was launched

**Figure 3:**
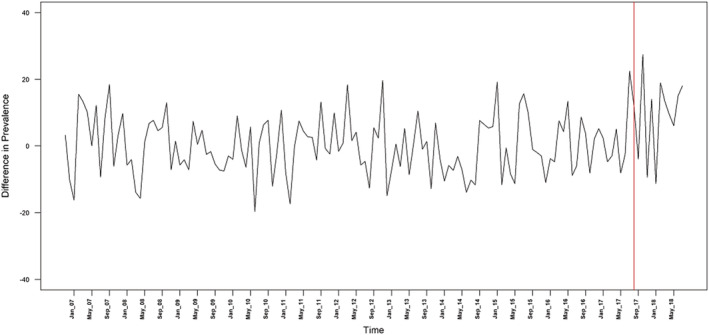
Difference in the prevalence of quit attempts between London and the rest of England. The red line denotes the time at which the intervention was launched

**Figure 4:**
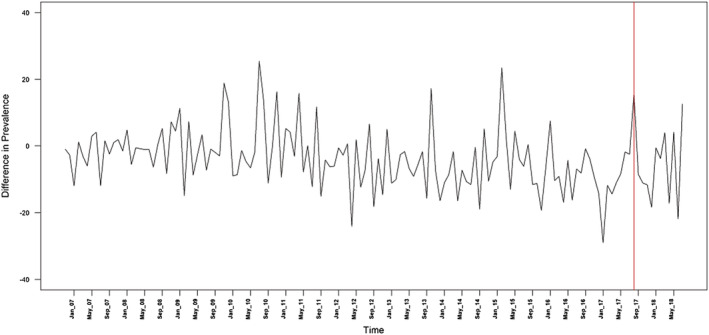
Difference in the success rate of quit attempts between London and the rest of England. The red line denotes the time at which the intervention was launched

Table 1 provides descriptive data on the mean monthly prevalence of quit attempts, use of cessation support and success of quit attempts. The mean monthly prevalence of quit attempts differed very little by region before the intervention (36.2% in London compared with 36.2% in the rest of England), but was higher in London compared with the rest of England after the intervention (39.0 versus 29.8%) (Fig. 1). The mean monthly prevalence of use of cessation support was slightly lower in London compared with the rest of England before (17.6 versus 19.8%) and after the intervention (7.3 versus 10.6%), as was the success rate of quit attempts (11.4 versus 16.7% before the intervention, 12.2 versus 17.9% after the intervention) (Fig. 2). Regional differences in the prevalence of quit attempts and the success rate of quit attempts over the time–series are shown in Figs. 3 and 4, respectively.

**Table 1:** Mean monthly prevalence of quit attempts, use of cessation support and quit success rates in London compared with the rest of England

	Mean (SD) overall	Mean (SD) before the intervention	Mean (SD) after the intervention
Monthly prevalence of quit attempts (12 months)			
London	36.47 (9.03)	36.24 (9.00)	38.98 (9.45)
Rest of England	35.66 (5.17)	36.20 (4.84)	29.82 (5.23)
Difference (London minus rest of England)	0.81 (9.34)	0.04 (8.73)	9.16 (11.82)
Monthly prevalence of use of cessation support^a^			
London	16.70 (12.03)	17.56 (12.11)	7.29 (5.43)
Rest of England	18.99 (6.18)	19.75 (5.83)	10.63 (2.95)
Monthly success rate of quit attempts			
London			
Imputation	13.02 (7.36)	12.98 (7.31)	13.23 (9.66)
No imputation	11.52 (8.49)	11.43 (8.48)	12.21 (10.40)
Rest of England	16.82 (5.10)	16.72 (4.92)	17.94 (6.92)
Difference (London minus rest of England)			
Imputation	−3.80 (9.33)	−3.74 (9.07)	−4.71 (12.16)
No imputation	−5.30 (10.11)	−5.29 (9.77)	−5.73 (13.82)

For London, prevalence in some months was zero and so values were imputed using Kalman smoothing for univariate time–series data [30].

^a^ Any use of behavioural support, prescription medication, telephone support or digital support in the most recent quit attempt among smokers who made a quit attempt in the past 12 months. SD = standard deviation.

Table 2 summarizes the results of the ARIMA models. There was a significant increase in the mean monthly difference in prevalence of quit attempts among smokers in London compared with smokers in the rest of England from 0.04 in the pre‐intervention period to 9.629 in the post‐intervention period. The monthly quit success rate was also higher, but the difference was not statistically significant. The Bayes factor was 1.21, which slightly favoured the hypothesis of an increase in quit success but showed that the data were insensitive.

**Table 2:** Results of the ARIMA models assessing the association between the implementation of the intervention and prevalence of attempts to quit smoking

	B	Lower CI	Upper CI	P
Mean difference in quit attempt prevalence (London minus rest of England)				
Model 1 no seasonal AR term	9.121	3.806	14.435	0.001
Model 2 seasonal AR term	9.589	4.346	14.833	< 0.001
Mean difference in success rate of quit attempts (London minus rest of England)				
Model 3 no seasonal AR term				
No imputation	4.129	−3.520	11.778	0.290
Imputation	3.510	−3.642	10.662	0.336
Model 4 seasonal AR term				
No imputation	4.716	−2.676	12.108	0.211
Imputation	3.618	−3.411	10.648	0.313

Model 1 = MA1, *P* < 0.001; model 2: MA1, *P* < 0.001, SAR1 *P* = 0.035; model 3: MA1, *P* < 0.001; model 4: MA1, *P* < 0.001, SAR1, *P* = 0.052. ARIMA = autoregressive integrated moving average; CI = confidence interval.

Supporting information, Fig. S1 shows the difference between the prevalence of quit attempts and quit success rates observed in the intervention period versus the figures forecast under the assumption of no intervention effect. This indicates that the monthly prevalence of quit attempts was higher in the post‐intervention period than predicted.

Table 3 shows the results of the GAM analyses assessing the interaction between region and a step‐level change in prevalence of quit attempts following the initiation of the intervention. There was a significant interaction between level (reflecting a step‐level change) and region (OR = 1.5, *P* < 0.001), indicating that the OR for the step‐level change was 1.5 times larger in London than in the rest of England. This effect remained after adjustment for age, sex and social grade.

**Table 3:** Results of the GAM model assessing the association between the implementation of the intervention and quit attempts

	OR	Lower CI	Upper CI	P
Unadjusted for sex, age and lower social grade				
Intercept	0.779	0.735	0.826	< 0.001
Trend	0.994	0.994	0.995	< 0.001
Level	1.169	1.039	1.315	0.009
Slope	0.997	0.994	0.999	0.004
Region	1.103	1.000	1.216	0.050
Trend × region	0.999	0.998	1.001	0.256
Level × region	1.518	1.206	1.910	< 0.001
Slope × region	1.003	0.998	1.008	0.247
Adjusted for sex, age and lower social grade				
Intercept	0.969	0.887	1.060	0.495
Trend	0.995	0.994	0.996	< 0.001
Level	1.160	1.030	1.306	0.014
Slope	0.997	0.994	0.999	0.006
Region	1.089	0.987	1.202	0.089
Trend × region	0.999	0.914	1.092	0.177
Level × region	1.567	1.550	1.584	< 0.001
Slope × region	1.002	0.966	1.040	0.368

Trend is the underlying trend in quit attempts prior to the intervention. Slope is the change in the underlying trend associated with the intervention. Trend was coded 1. 141, level 0 before the intervention and 1 after the intervention, slope was coded 0 before the intervention and 1. 12 following the intervention and regions was coded 0 for the control region and 1 for London. GAM = generalized additive models; OR = odds ratio; CI = confidence interval.

Table 4 shows the results of the GAM analysis assessing the interaction between region and a step‐level change in prevalence of the success of quit attempts following initiation of the intervention. No significant interaction was identified and the data were insensitive (BF = 1.06).

**Table 4:** Results of the GAM model assessing the association between the implementation of the intervention and success of quit attempts

	OR	Lower CI	Upper CI	P
Unadjusted for sex, age and lower social grade				
Intercept	0.158	0.141	0.176	< 0.001
Trend	1.003	1.001	1.004	0.004
Level	1.014	0.800	1.287	0.906
Slope	0.993	0.986	1.001	0.069
Region	0.837	0.662	1.058	0.136
Trend × region	0.996	0.992	0.999	0.012
Level × region	1.215	0.705	2.095	0.483
Slope × region	1.010	0.998	1.022	0.120
Adjusted for sex, age and lower social grade				
Intercept	0.189	0.158	0.227	< 0.001
Trend	1.002	1.001	1.004	0.007
Level	1.007	0.797	1.273	0.951
Slope	0.993	0.986	1.001	0.073
Region	0.811	0.641	1.025	0.080
Trend × region	0.996	0.992	0.999	0.016
Level × region	1.212	0.702	2.092	0.490
Slope × region	1.009	0.997	1.022	0.126

Trend is the underlying trend in success of quit attempts prior to the intervention. Slope is the change in the underlying trend associated with the intervention. Trend was coded 1. 141, level 0 before the intervention and 1 after the intervention, slope was coded 0 before the intervention and 1. 12 following the intervention, and regions was coded 0 for the control region and 1 for London. GAM = generalized additive models; OR = odds ratio; CI = confidence interval.

Results from an unplanned analysis of past‐month quit attempts among smokers were similar to those for past 12‐month quit attempts (Supporting information, Tables 1–3), with a significant increase in the mean monthly difference in quit attempts between smokers in London versus the rest of England in the ARIMA analysis and a significant interaction between level and region in the GAM analysis. Results from an exploratory, unplanned analysis of overall quits among all smokers were similar to those for quit success among smokers who tried to stop (Supporting information, Tables 4–6), with a slightly higher quit rate in London versus the rest of England that did not reach statistical significance. Data favoured the hypothesis of an increase in quits but were insensitive (BF = 1.05–1.12).

## Discussion

The first year of the London Smoking Cessation Transformation Programme, a multi‐component smoking cessation intervention in September 2017, was associated with an increase in the regional prevalence of quit attempts relative to the rest of England. The mean success rate among smokers who tried to quit was slightly but not significantly higher.

The present findings are in line with previous studies that have found benefits of national social marketing campaigns to promote smoking cessation. For example, a large‐scale evaluation of England's national ‘Stoptober' smoking cessation campaign, which encourages smokers to be smoke‐free for the month of October, saw a significant increase in the rate of quit attempts during October compared with other months of the same year, with the odds of a smoker trying to quit increasing by 50% [18]. They also parallel results of city‐wide campaigns conducted in the United States and Australia. In New York City, a programme offering free nicotine patches to smokers interested in quitting via a Quitline produced a significant increase in quit attempts and cessation over 12 months [41]. In a study across five cities in California with a 6‐year follow‐up, a significantly higher rate of quitting was observed in cities where a community‐based smoking cessation campaign was run compared with control cities [42]. Similarly, in Sydney, the city‐wide quit rate in the 12 months following a media‐based campaign ‘Quit. For Life' was significantly higher than was observed over the same period in Melbourne, where no such campaign was run [43]. Our findings are also consistent with evidence collected in health‐care settings that has shown that directing smokers to cessation support can increase the rate of quit attempts. A meta‐analysis of 13 randomized controlled trials found that a brief offer of pharmacological or behavioural support for cessation from a physician was associated with a significantly higher rate of quit attempts among smokers compared with no intervention or advice to quit on medical grounds, although evidence for an effect on quit success was inconclusive [44]. Taken together, these results suggest that an offer of support is a key strategy for promoting quit attempts, but other factors may be more relevant to quit success. The findings have direct and actionable implications: the LSCTP and its promotion warrant continued investment to promote quit attempts in London.

A striking result was that the monthly prevalence of quit attempts fell substantially from pre‐ to post‐intervention in the rest of England (from 36.2 to 29.8%)—which might be expected in the context of reduced national mass media budgets and local public health budget cuts—but increased in London. Use of cessation support declined in both regions, emphasizing the need for a reversal of cuts to stop smoking service budgets nation‐wide [6].

The study strengths include the large, representative sample of adult smokers across all regions of England, a long baseline time–series prior to implementation of the programme and monthly data collection. Obtaining consistent results from two different statistical modelling approaches strengthens confidence in the findings.

There were several limitations to the study. First, all data were from self‐reports, introducing scope for error and bias. However, in population studies the social pressure to misreport smoking status is low [45]. Secondly, the measure of exposure (lives in London versus lives elsewhere) may not accurately distinguish between participants who were and were not exposed to the campaign. It is common for people who work in London to commute from neighbouring regions, which may have led to contamination of the control group. If exposure to the campaign encouraged people living in other regions to make a quit attempt or access local stop smoking services, then this contamination would have limited the statistical power to detect what may have been meaningful increases in success rates. Thirdly, this study only examined quit attempts and quit success rates. There were insufficient data to formally assess whether there had been a relative increase in use of stop‐smoking support in London compared with the rest of England, but descriptive pre‐ and post‐intervention data were not suggestive of a differential regional change in use of cessation support over time. It is possible that much or all the impact of the intervention was the result of the social marketing campaign. An independent review of the pilot stage of the LSCTP indicated that the marketing supported uptake of the on‐line portal but not the helpline (see Supporting information). Whether this was true of the remainder of the study period is not known. Fourthly, the outcome measures relied on quit attempts in the last year rather than the last month, which meant the assessment period after the intervention would not have related exclusively to the intervention. ARIMA analyses are designed to model such noise, and a sensitivity analysis of past‐month quit attempts showed a similar pattern of results. Fifthly, the GAM analysis only modelled linear trends. Sixthly, while the intervention campaign was launched in September 2017, some of the components of the programme were available from May 2017 and we did not model this initial intervention period. Finally, we cannot rule out the possibility that an unmeasured confounding factor led to the increase in quit attempts in London.

In conclusion, the first year of the London Smoking Cessation Transformation Programme, a multi‐component smoking cessation programme, was associated with a significant increase in prevalence of quit attempts in London compared with the rest of England. Evidence for an impact on the success of quit attempts was inconclusive.

## Declaration of interests

J.B. and E.B. have received unrestricted research funding from Pfizer, who manufacture smoking cessation medications. R.W. undertakes research and consultancy for and receives travel funds and hospitality from manufacturers of smoking cessation medications (Pfizer, GlaxoSmithKline and Johnson and Johnson). All authors declare no financial links with tobacco companies or e‐cigarette manufacturers or their representatives. S.E.J. affirms that the manuscript is an honest, accurate, and transparent account of the study being reported; that no important aspects of the study have been omitted; and that any discrepancies from the study as originally planned (and registered) have been explained.

## Author contributions

**Sarah Jackson:** Conceptualization; investigation; methodology. **Emma Beard:** Conceptualization; data curation; formal analysis; investigation; methodology; visualization. **Robert West:** Conceptualization; data curation; funding acquisition; investigation; methodology; supervision. **Jamie Brown:** Conceptualization; data curation; funding acquisition; investigation; methodology; supervision.

## Supporting information


**Figure S1.** Actual and forecasted values (assuming the intervention had not taken place) for a) prevalence of quit attempts and b) prevalence of the success of quit attempts. Note: Both graphs are based on the ARIMAX models with seasonal MA terms; imputed time series is used for the prevalence of successful quit attempt.
**Table S1.** Results of the ARIMA models assessing the association between the implementation of the intervention and prevalence of attempts to quit smoking in the past month
**Table S2.** Prevalence of quit attempts in the past month used in the GAM analysis
**Table S3.** Results of the GAM model assessing the association between the implementation of the intervention and quit attempts in the past month
**Table S4.** Results of the ARIMA models assessing the association between the implementation of the intervention and prevalence of overall quits
**Table S5.** Prevalence of overall quit rates used in the GAM analysis
**Table S6.** Results of the GAM model assessing the association between the implementation of the intervention and overall quits.
