# Supplementary material for: Evaluation of the London Smoking Cessation Transformation Programme: a time–series analysis
Source: Addiction. 2020 Dec 29;116(6):1558–68. doi: 10.1111/add.15367 (PMC8247014; doi:10.1111/add.15367)
Supplement: Supplementary file 1 — Figure S1. Actual and forecasted values (assuming the intervention had not taken place) for a) prevalence of quit attempts and b) prevalence of the success of quit attempts. Note: Both graphs are based on the ARIMAX models with seasonal MA terms; imputed time series is used for the prevalence of successful quit attempt. Table S1. Results of the ARIMA models assessing the association between the implementation of the intervention and prevalence of attempts to quit smoking in the past month Table S2. Prevalence of quit attempts in the past month used in the GAM analysis Table S3. Results of the GAM model assessing the association between the implementation of the intervention and quit attempts in the past month Table S4. Results of the ARIMA models assessing the association between the implementation of the intervention and prevalence of overall quits Table S5. Prevalence of overall quit rates used in the GAM analysis Table S6. Results of the GAM model assessing the association between the implementation of the intervention and overall quits. [file ADD-116-1558-s001.docx]

**Further details of the London Smoking Cessation Transformation Programme**

The London Smoking Cessation Transformation Programme (LSCTP) was established as a collaborative across London local authorities in 2016. At its outset, the Programme aim was to support London boroughs to transform and improve the way their residents accessed stop smoking support, with the potential to deliver savings to participating local authorities and improved outcomes for their residents. The scope of the Programme was to seek to offer boroughs innovative service options in alternative channels (digital, online, telephone) and which could be delivered once across London, to complement locally-determined services such as face to face models. The Programme presented an opportunity to learn together at scale and pace what works, and to jointly commission where it made sense to do so.

The Programme is overseen by a Programme Board with representation including Directors of Public Health or their deputies from each of the five London sub-regions. The Programme and its administrative office is hosted by Public Health, London Borough of Tower Hamlets.

Thirty-one London boroughs jointly commission and participate in the LSCTP which is now part of a broader stop smoking and tobacco control landscape that includes education, regulation and enforcement. The LSCTP remains focused on reducing the prevalence of smoking in London and works to:

1. Improve public awareness, access to and therefore uptake of all Stop Smoking Services.
2. Continue to build upon the Programme’s asset base and innovate services, creating greater synergy between regional and local Stop Smoking Services.
3. Develop and provide an evidence based Stop Smoking London Service reflecting the needs and lifestyles of London smokers.
4. Create a Stop Smoking social movement across London that supports smokers to quit permanently.

**PHASE 1:** To meet the Programme aim, a pilot (phase 1) marketing and communications campaign to direct smokers to a London wide Stop Smoking helpline ran from May 2017- March 2018. In the summer of 2017 the Programme agreed to work alongside Professor Robert West and utilise an existing ‘Stop Smoking London Portal’.

The helpline is provided by NHS Smokefree and can be reached on 0300 123 1044. It provides a convenient way for Londoners to access good quality, specialist advice on how to quit smoking. It’s also a gateway to finding out more about the range of free and varied support available across the capital. Smokers can call the local rate number seven days a week – Monday to Friday from 9am to 8pm and on Saturday and Sunday between 11am and 4pm. Callers are able to speak to a professionally trained advisor to find out what support is available in their area and establish which quit methods will work best, from different types of medication, to mobile apps and specialist programmes. When smokers call the helpline, they are asked a series of questions to determine the level of stop smoking support they need. If they are from London and meet the defined participation criteria, they are offered the opportunity to sign up to the specialist telephone-based service. This service provides a proactive four-week behavioural support intervention.

The online portal provides evidence-based information about the reasons for smoking, tools and resources to help smokers to quit, local support services, and the benefits of stopping smoking in an interactive, user-friendly format. A smoking calculator allows users to calculate the amount of money they could save by quitting. The helpline number is featured prominently at the top of each page, and users can search by borough or postcode to locate and access local support.

A phased implementation of a public facing marketing and advertising campaign launched in September 2017. This included radio, public realm and online advertising of both the web Portal and helpline and utilised existing national campaign opportunities, including Stoptober and No Smoking Day.

The pilot (phase 1) completed on time and as planned in March 2018. At this time, the LSCTP Board commissioned an independent review to evaluate the pilot, which sought to answer the following questions:

1. How did the different facets of the Stop Smoking London Services interact during the pilot?
2. What effect did the promotional campaign play during the pilot?
3. What was the performance of the helpline and what contributions were made by the London Stop Smoking Portal during the pilot?
4. What were the views from stakeholders of the pilot?
5. Moving forward what learning could we take from the pilot and from other public health initiatives?

Based on evaluation of data between September 2017 and March 2018, the review highlighted the following:

1. Marketing is supporting the web Portal. It amassed ~2 million impressions and in excess of 43,000 click-throughs to the Portal. Half of the total visitors to the Portal landing pages chose to explore local service options and sought further information via the Portal. Nearly 500 people registered their email address to receive updates on Stop Smoking London.
2. However, marketing does not seem to be supporting the uptake of the helpline, with only a small number of visits to the landing pages of the web Portal leading to calls to the helpline (*n*=66). This may be because it is not targeting the right people in the right place or indeed in the right way.
3. Over 400 direct calls were made to the London helpline between September 2017 and March 2018. Not all callers were eligible for the service (i.e. pregnant, not a London resident, etc.) – 365 out of 472 London callers were eligible. The number of people calling the helpline increased with marketing activity.
4. Importantly, once smokers were engaged with the stop smoking helpline, four-week proactive behavioural support and are contactable, the quit rate was 76.5%. This is in keeping with the Russell Standard (Clinical), which states the self-reported four-week success rate should generally be over 50% (1).
5. Hearing about the service, largely through digital realms, to an intimate and personal conversation with a remote health advisor; may have been too great a channel shift for service users and contributed to the lower than anticipated take-up.
6. Confusion around the roles of the website and the phone service and perceived competition with local services may have contributed to differences in the way the London telephone service and web Portal were publicised locally.
7. It is possible that continuing to focus attention solely on a telephone helpline, website and supporting communications programme is too prescriptive in scope.
8. There is some relevant experience to be had from other initiatives around the UK and the LSCTP should join up and share information going forward.
9. There is energy and good will across London stakeholders for a coherent smoking cessation offer.

More information on the results of the pilot can be found in the slide set “LSCTP programme journey and way forward”, which is located on the ADPH London Smoking cessation webpage (http://adph.org.uk/networks/london/programme/smoking-cessation/).

**PHASE 2:**The results of the review have allowed the LSCTP to identify opportunities to build on and strengthen current work to achieve the aim of improving access and uptake of Stop Smoking Services across London.

In light of the review the Programme’s current objectives are to:

1. Remain focused on reducing the prevalence of smoking in London.
2. Continue to improve public awareness, access to and therefore uptake of all Stop Smoking Services.
3. Continue to build upon the Programme’s asset base and innovate services, creating greater synergy between regional and local Stop Smoking Services.
4. Develop and provide an evidence based Stop Smoking London Service reflecting the needs and lifestyles of London smokers.
5. Create a Stop Smoking social movement across London that supports smokers to quit permanently.

**Phase 2 - Stop Smoking London Website**

A new Stop Smoking London website (https://www.stopsmokinglondon.com/) been created, which gives people a range of tools they can use via their phone. Work is also underway exploring ways to build on this further to make it a real asset for Londoners.

**Phase 2 - Stop Smoking London Marketing and Advertising Campaign**

**Amazing Things Happen Campaign**

This campaign, which will run continuously up until 31^st^ March 2019, is specifically targeted at London smokers aged 25 – 40 years old, the largest smoking population in the UK. The campaign is designed to encourage London Smokers to engage with both local and regional support in order to set a quit date and start their supported stop smoking journey.

Further information can be found at http://adph.org.uk/networks/london/programme/smoking-cessation/.

**Further details on ARIMA modelling procedures**

Standard recommended procedures (2,3) were used to select the ARIMA models. First, each time series was assessed for outlying values that may bias the results using a procedure described in Chen and Liu (4). This involved fitting a loess curve and identifying residuals as outliers if they were outside the range $\pm2\left( q_{0.9}-q_{0.1} \right),$ where the $q_{p}$ is the p-quartile of the residuals. No outliers were identified. Secondly, the plots of the differenced data and unit root tests (i.e. Osborn-Chui-Smith-Birchenhall test and Kwiatkowski, Phillips, Schmidt, and Shin (KPSS) test) were used to determine the number of seasonal and non-seasonal differences required for the time series to be stationary (5,6). One order of differencing was required for all analyses. Thirdly, to determine the initial values of the AR and MA terms for the baseline models, the autocorrelation function (ACF) and partial autocorrelation function (PACF) were assessed. Additional models with various fitted AR and MA terms were then compared to this baseline model using the Akaike information criterion (AIC). According to the Box-Jenkins method, we only checked ARIMA models for p and q values of 3 or less (2). These suggested that a model with one MA term provided the best fit. Thus the best fitting model for the analyses was an ARIMA(0,1,1)_12_ i.e. a model with one order of differencing and one non-seasonal MA term.

Finally, the Ljung-Box test for white noise and the ACF for the residuals of the best fitting models were checked for additional correlation (thus the need for additional MA/AR seasonal or non-seasonal terms) and the coefficients of the correlation terms assessed for significance and whether they fell within the bounds of stationarity and invertibility (7,8). The residuals for all of the models were not free of serial correlation (9). This was amended with the addition of a seasonal AR term, i.e. a seasonal ARIMA(0,1,1)(1,0,0)_12_. A sensitivity analysis was conducted which forecast the predicted prevalence of quit attempts and quit success from September 2017 using the pre-intervention data. This gives the predicted values for September 2017 until August 2018 assuming no intervention had taken place.

**References**

1. West R. Assessing smoking cessation performance in NHS Stop Smoking Services: The Russell Standard (Clinical) [Internet]. 2005 [cited 2019 Jun 3]. Available from: http://www.ncsct.co.uk/usr/pub/assessing-smoking-cessation-performance-in-nhs-stop-smoking-services-the-russell-standard-clinical.pdf

2. Box GE, Jenkins GM, Reinsel GC, Ljung GM. Time series analysis: forecasting and control. John Wiley & Sons; 2015.

3. Box GEP, Tiao GC. Intervention Analysis with Applications to Economic and Environmental Problems. J Am Stat Assoc. 1975;70(349):70–9.

4. López-de-Lacalle J. tsoutliers R Package for Detection of Outliers in Time Series. In 2016.

5. Lee D, Schmidt P. On the power of the KPSS test of stationarity against fractionally-integrated alternatives. J Econom. 1996 Jul 1;73(1):285–302.

6. Osborn DR. Seasonality and the Order of Integration for Consumption. Oxf Bull Econ Stat. 1988;50(4):361–77.

7. Yaffee RA, McGee M. An Introduction to Time Series Analysis and Forecasting: With Applications of SAS® and SPSS®. San Diego: Academic Press; 2000. 528 p.

8. Yaffee RA. An Introduction to Forecasting Time Series with Stata. Taylor and Francis; 2012.

9. Montgomery DC, Jennings CL, Kulahci M. Introduction to Time Series Analysis and Forecasting. 2nd edition. Hoboken, New Jersey: Wiley-Blackwell; 2015. 672 p.


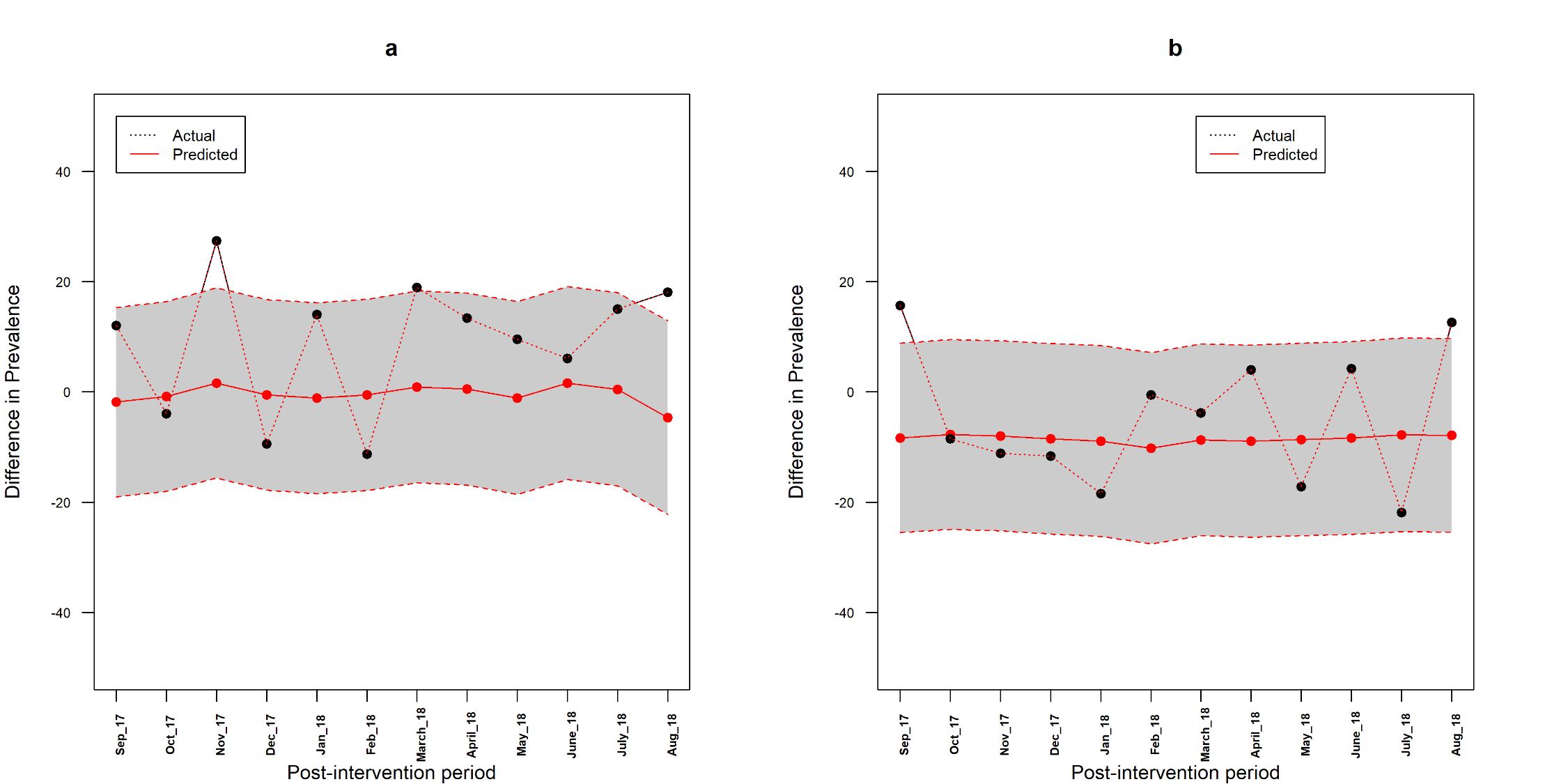
**Supplementary Figure 1:** Actual and forecasted values (assuming the intervention had not taken place) for a) prevalence of quit attempts and b) prevalence of the success of quit attempts. Note: Both graphs are based on the ARIMAX models with seasonal MA terms; imputed time series is used for the prevalence of successful quit attempt.

**Supplementary Table 1:** Results of the ARIMA models assessing the association between the implementation of the intervention and prevalence of attempts to quit smoking in the past month

|  | B | Lower CI | Upper CI | P |
| --- | --- | --- | --- | --- |
| **Mean difference in overall quits (London minus Rest of England)**  *Model 1 no seasonal AR term*  *Model 2 seasonal AR term* | 3.176  3.044 | 0.319  0.234 | 6.032  5.853 | 0.029  0.034 |

**Supplementary Table 2:** Prevalence of quit attempts in the past month used in the GAM analysis

|  | Mean (SD) overall | Mean (SD) before the intervention | Mean (SD) after the intervention |
| --- | --- | --- | --- |
| Quit attempts in the past month  *London*  *Rest of England* | 8.54 (4.10)  6.84 (1.83) | 8.48 (4.01)  6.98 (1.79) | 9.28 (5.14)  5.43 (1.73) |

**Supplementary Table 3:** Results of the GAM model assessing the association between the implementation of the intervention and quit attempts in the past month

|  | OR | Lower CI | Upper CI | P |
| --- | --- | --- | --- | --- |
| Unadjusted |  |  |  |  |
| Intercept | 0.094 | 0.084 | 0.104 | <0.001 |
| Trend | 0.996 | 0.994 | 0.998 | <0.001 |
| Level | 1.059 | 0.846 | 1.326 | 0.617 |
| Slope | 1.002 | 0.998 | 1.006 | 0.242 |
| Region | 1.534 | 1.301 | 1.809 | <0.001 |
| Trend*region | 0.997 | 0.995 | 1.000 | 0.027 |
| Level*region | 1.547 | 1.044 | 2.291 | 0.030 |
| Slope*region | 1.005 | 0.998 | 1.011 | 0.191 |
| Adjusted for sex, age and lower social-grade |  |  |  |  |
| Intercept | 0.123 | 0.104 | 0.144 | <0.001 |
| Trend | 0.996 | 0.995 | 0.998 | <0.001 |
| Level | 1.048 | 0.837 | 1.313 | 0.682 |
| Slope | 1.002 | 0.999 | 1.006 | 0.227 |
| Region | 1.514 | 1.283 | 1.786 | <0.001 |
| Trend*region | 0.997 | 0.995 | 1.000 | 0.021 |
| Level*region | 1.579 | 1.065 | 2.341 | 0.023 |
| Slope*region | 1.004 | 0.997 | 1.011 | 0.218 |

Note: Trend is the underlying trend in quit attempts. Slope is the change in trend associated with the intervention. Trend was coded 1 . . . 141, level 0 before the intervention and 1 after the intervention, slope was coded 0 before the intervention and 1 . . . 12 following the intervention, and regions was coded 0 for the control region and 1 for London.

**Supplementary Table 4:** Results of the ARIMA models assessing the association between the implementation of the intervention and prevalence of overall quits

|  | B | Lower CI | Upper CI | P |
| --- | --- | --- | --- | --- |
| **Mean difference in overall quits (London minus Rest of England)**  *Model 1 no seasonal AR term*  No imputation  Imputation  *Model 2 seasonal AR term*  No imputation  Imputation | 0.660  0.762  0.627  0.770 | -2.148  -1.944  -2.166  -1.950 | 3.468  3.468  3.420  3.491 | 0.645  0.581  0.660  0.579 |

**Supplementary Table 5:** Prevalence of overall quit rates used in the GAM analysis

|  | Mean (SD) overall | Mean (SD) before the intervention | Mean (SD) after the intervention |
| --- | --- | --- | --- |
| Overall quits  *London*  *Rest of England* | 5.06 (3.27)  6.08 (1.82) | 5.05 (3.34)  6.10 (1.78) | 5.08 (2.51)  5.91 (2.34) |

**Supplementary Table 6:** Results of the GAM model assessing the association between the implementation of the intervention and overall quits

|  | OR | Lower CI | Upper CI | P |
| --- | --- | --- | --- | --- |
| Unadjusted |  |  |  |  |
| Intercept | 0.065 | 0.058 | 0.073 | <0.001 |
| Trend | 0.999 | 0.997 | 1.001 | 0.186 |
| Level | 1.141 | 0.912 | 1.427 | 0.247 |
| Slope | 0.944 | 0.989 | 1.000 | 0.048 |
| Region | 0.897 | 0.717 | 1.122 | 0.341 |
| Trend*region | 0.996 | 0.993 | 0.999 | 0.013 |
| Level*region | 1.460 | 0.886 | 2.407 | 0.137 |
| Slope*region | 1.008 | 0.997 | 1.018 | 0.169 |
| Adjusted for sex, age and lower social-grade |  |  |  |  |
| Intercept | 0.089 | 0.075 | 0.107 | <0.001 |
| Trend | 0.999 | 0.997 | 1.000 | 0.109 |
| Level | 1.139 | 0.911 | 1.424 | 0.254 |
| Slope | 0.995 | 0.989 | 1.000 | 0.054 |
| Region | 0.860 | 0.687 | 1.077 | 0.188 |
| Trend*region | 0.996 | 0.993 | 0.999 | 0.019 |
| Level*region | 1.447 | 0.878 | 2.386 | 0.147 |
| Slope*region | 1.007 | 0.996 | 1.018 | 0.205 |
